# Heat-Inactivated *Lacticaseibacillus paracasei* PC-01 Alleviates DSS-Induced Colitis in Mice by Modulating Gut Microbiota and Intestinal Barrier Function

**DOI:** 10.3390/nu18193199

**Published:** 2026-09-28

**Authors:** Xinxue Zhao, Chang Yang, Lu Gao, Yinyin Gao, Wentao Qian, Hongxia Liu, Zhihong Sun, Guohong Wang, Feiyan Zhao

**Affiliations:** 1 Key Laboratory of Dairy Biotechnology and Engineering, Ministry of Education, Inner Mongolia Agricultural University, Hohhot 010018, China; 2 Key Laboratory of Dairy Products Processing, Ministry of Agriculture and Rural Affairs, Inner Mongolia Key Laboratory of Dairy Biotechnology and Engineering, Hohhot 010018, China; 3 Inner Mongolia Mengniu Dairy (Group) Co., Ltd., Hohhot 011500, China; 4 Mengniu Hi-Tech Dairy Products (Beijing) Co., Ltd., Beijing 101100, China

**Keywords:** inflammatory bowel disease, *Lacticaseibacillus paracasei* PC-01, gut microbiota, barrier function

## Abstract

**Background:** Inflammatory bowel disease (IBD) is a chronic, progressive, immune-mediated inflammatory disorder of the gastrointestinal tract. **Methods:** This study used C57BL/6J mice induced by DSS to construct an intestinal inflammation model, and explored the alleviating effect and potential mechanism of heat inactivated *Lactobacillus paracasei* PC-01 (PC-01) on intestinal inflammation. **Results:** The results showed that heat-inactivated PC-01 significantly decreased the Disease Activity Index and H&E histopathological scores (*p* < 0.05). It also reduced the serum levels of pro-inflammatory cytokines, including IL-6, IL-1β, TNF-α, and malondialdehyde (*p* < 0.01), increased the levels of the anti-inflammatory factor IL-10 (*p* < 0.001) and antioxidant enzymes SOD and GSH-Px (*p* < 0.01), and restored the integrity of the intestinal epithelial barrier in DSS-induced colitis mice. Metagenomic analysis showed that PC-01 treatment restored the abundance of beneficial bacteria, including *Barnesiella* and *Muribaculum*, and reduced the enrichment of opportunistic pathogens such as *Angelakisella* and *Lawsonibacter* (*p* < 0.05). Further functional annotation of the microbial community indicated that PC-01 significantly enriched pathways related to the TCA cycle, riboflavin metabolism, Toll and Imd signaling, and lipopolysaccharide biosynthesis. In addition, the relative abundances of auxiliary activities, polysaccharide lyases, and S-layer homology modules were lower than those in the DSS group, whereas those of glycoside hydrolases, glycosyltransferases, and carbohydrate esterases were higher. At the transcriptional level, intervention with heat-inactivated PC-01 resulted in a downward trend in the expression of TLR4, Nfkb1, and Rela, accompanied by reduced expression of the chemokines MCP-1 and Cxcl10, and inflammasome-related genes NLRP3, Casp1, and IL-1β. **Conclusions:** In summary, heat-inactivated *Lacticaseibacillus paracasei* PC-01 effectively alleviated intestinal inflammatory injury in mice by regulating gut microbiota homeostasis and suppressing intestinal inflammatory responses.

## 1. Introduction

Inflammatory bowel disease (IBD) is a chronic, progressive, immune-mediated disorder characterized by persistent inflammation of the gastrointestinal tract [1]. IBD comprises two major clinical entities: Crohn’s disease (CD) and ulcerative colitis (UC) [2]. Epidemiological evidence indicates that approximately 6–8 million people are affected by IBD worldwide. Although the incidence of IBD has plateaued in developed Western countries, it continues to rise in newly industrialized regions [3]. By 2030, the prevalence of IBD in several early industrialized countries is projected to reach 1% of the population [4]. Current treatment options, including biologics and immunosuppressants, can improve disease control; however, their long-term use is often limited by adverse effects and high costs, which restrict their broader clinical application [5].

The onset and progression of IBD involve complex multifactorial pathological processes. Its pathogenesis is associated with genetic susceptibility, environmental influences, gut microbiota dysbiosis, and excessive immune activation [6]. Among these factors, gut microbiota imbalance, aberrant immune responses, and intestinal barrier impairment are considered central features of the disease [7]. Previous studies have shown that patients with IBD exhibit marked disturbances in gut microbial composition during inflammation, including a substantial reduction in beneficial butyrate-producing bacteria, such as *Roseburia hominis* and *Faecalibacterium prausnitzii*, and an increase in facultative anaerobes, such as *Escherichia coli* [8]. Gut microbiota dysbiosis can directly contribute to intestinal dysfunction and immune activation, thereby disrupting epithelial tight junction integrity and aggravating inflammation [9]. One study used fecal microbiota transplantation (FMT) to restore gut microbial homeostasis in mice and found that it reduced the Disease Activity Index (DAI) scores, increased body weight and colon weight and length, markedly alleviated colonic pathological injury, increased the relative abundance of Firmicutes, and reduced the abundance of Bacteroidetes and Proteobacteria. These findings suggest that modulation of the gut microbiota can effectively ameliorate IBD-related symptoms and further support the gut microbiota as an important target for IBD intervention [10].

Probiotics are microorganisms that confer beneficial effects on host health when administered in adequate amounts [11]. They have attracted considerable attention because of their potential to regulate gut microbiota and alleviate gastrointestinal disorders, including functional constipation and irritable bowel syndrome [12]. Pang et al. reported that *Lacticaseibacillus rhamnosus* MP108 significantly increased weekly bowel frequency and improved Bristol stool scores by modulating the gut microbiota [13]. However, live probiotics have important limitations in their practical applications. Their viability and stability can be readily affected by storage, processing, and gastrointestinal transit, potentially compromising their efficacy [14]. Increasing evidence suggests that heat-inactivated probiotics may have effects comparable to or even greater than those of live bacteria in regulating gut microbiota and improving disease symptoms. Clara et al. found that heat-inactivated *Akkermansia muciniphila* increased insulin sensitivity by approximately 30%, reduced insulinemia and plasma total cholesterol, and showed better efficacy than the live form. In addition, inactivated bacteria are safer, more stable, and less susceptible to inactivation [15]. Therefore, heat-inactivated probiotics are increasingly being explored as a safer and more clearly defined strategy for gut microbiota regulation. Compared to live probiotics, heat-inactivated preparations offer practical advantages, including better stability and easier storage and transport [16].

In recent years, heat-inactivated probiotics have shown promising potential for the regulation of intestinal barrier function and host immune responses. Lisa et al. found that three heat-inactivated strains, including *Lactobacillus acidophilus* PM-A0002, *Lactobacillus gasseri* PM-A0005, and *Lactobacillus saliVarius* PM-A0006, exerted immunomodulatory effects and enhanced intestinal barrier integrity [17]. Heat-inactivated *Lactobacillus acidophilus* PIN7 alleviates DSS-induced ulcerative colitis by modulating Toll-like receptor 6 (TLR) signaling and maintaining gut microbiota balance [18]. In addition, heat-sterilized *Lactobacillus plantarum* Zhang-LL alleviated ulcerative colitis symptoms by reducing pro-inflammatory cytokine levels, decreasing intestinal permeability by increasing the expression of tight junction-related proteins, including ZO-1, Claudin-1, and Occludin, and regulating the gut microbiota [19]. These findings suggest that probiotic inactivation may be a practical and effective strategy for the treatment of colitis. Notably, both heat-inactivated and live probiotics exhibit strain specificity, meaning that different strains may differ substantially in the active components released after inactivation and their immunoregulatory capacities [20]. Therefore, it is necessary to investigate the specific effects and mechanisms of individual heat-inactivated probiotic strains.

*Lacticaseibacillus paracasei* PC-01 (PC-01) is a probiotic strain isolated from naturally fermented sour yak milk in Lhasa, China [21]. Previous studies have shown that live PC-01 significantly ameliorates DSS-induced colonic lesions in mice and reduces serum inflammatory levels [22]. However, the anti-inflammatory effects of heat-inactivated PC-01 and its regulatory roles in microbiota homeostasis and mucosal barrier function during intestinal inflammation remain unclear. Accordingly, the present study established a DSS-induced mouse colitis model and systematically evaluated the protective effects and potential mechanisms of heat-inactivated PC-01 using metagenomic sequencing, enzyme-linked immunosorbent assay (ELISA), and other methods. In addition, this study aimed to provide a scientific basis for the functional development and application of heat-inactivated PC-01 as a postbiotic ingredient, particularly in ambient-stable dairy products, and to offer a methodological reference for the development and utilization of inactivated probiotic strains in the future.

## 2. Materials and Methods

### 2.1. Laboratory Animals

Six-week-old male C57BL/6J mice weighing 20–23 g were purchased from Beijing Huafukang Biotechnology Co., Ltd. (Beijing, China). Throughout the experiment, all animals were housed in a specific pathogen-free barrier facility under a 12 h light/dark cycle at an ambient temperature of 22 ± 2 °C and ambient humidity of 45 ± 10%. The mice were given ad libitum access to food and water.

Animal experiments were designed based on a DSS-induced colitis model, and the experimental workflow is shown in Figure 1A.

The sample size (n = 8 per group) was determined based on similar studies in the literature [23] that reported comparable experimental designs and outcome measures. After 1 week of acclimatization, 24 mice were randomly divided into three groups, with eight mice in each group: the PC-01 heat-inactivated bacterial group (DSS induction + oral gavage of heat-inactivated PC-01 bacterial suspension, 0.2 mL/day, containing 4 × 10^8^ cells/mL; PC-01 group), the blank control group (free diet + oral gavage of an equal volume of normal saline, 0.2 mL/day; NC group), and the model group (DSS induction + oral gavage of an equal volume of normal saline, 0.2 mL/day; DSS group). The rationale for this was to avoid cage-mate stress. Colitis was induced in the DSS and PC-01 groups using a 2.5% DSS solution. The mice in the NC group received sterile water ad libitum throughout the 30-day experimental period. Mice in the DSS and PC-01 groups received 2.5% DSS from days 0–5, followed by sterile water from days 5–10 for a total of three cycles to induce intestinal inflammation. Body weights were recorded and fecal samples were collected on days 0 and 30. Colitis severity was evaluated on days 5, 10, 15, 20, 25, and 30. On day 30, all mice were anesthetized by intraperitoneal injection of pentobarbital sodium (30 mg/kg) and euthanized by cervical dislocation. Colon morphology was recorded, and serum supernatant and colon tissue samples were collected for further analysis. No animals were excluded from the analysis in this experiment due to poor health or experimental manipulation errors. All experimental animals and data points were recorded and used for the evaluation of the results and statistical analysis.

### 2.2. Animal Care and Ethics Approval

The entire set of experimental procedures was formally reviewed and approved by the Institutional Animal Care and Use Committee (ACUC) at Inner Mongolia Agricultural University (Approval Number NND2025165, 25 April 2025).

### 2.3. Experimental Strains and Reagents

The strain used in this study was *Lacticaseibacillus paracasei* PC-01, provided by the Key Laboratory of Dairy Biotechnology and Engineering, Inner Mongolia Agricultural University, Ministry of Education, China. The strain was heat-inactivated in a 75 °C water bath for 15 min.

Mouse interleukin-6 (IL-6), interleukin-10 (IL-10), interleukin-1β (IL-1β), tumor necrosis factor-α (TNF-α), superoxide dismutase (SOD), and glutathione peroxidase (GSH-Px) ELISA kits, as well as the malondialdehyde (MDA) assay kit, were purchased from Nanjing Boyan Biotechnology Co., Ltd. (Nanjing, China). Antibodies against the tight junction proteins ZO-1, Occludin, and Claudin, as well as mucin 2 (MUC2), were purchased from Wuhan Sanying Biotechnology Co., Ltd. (Wuhan, China).

### 2.4. Colitis Severity Scoring

The DAI is a composite scoring system based on three indicators: body weight changes, stool consistency, and bleeding [24]. Specifically, the rate of body weight loss was calculated as [(W − W_0_)/W_0_ × 100%]. Stool consistency was scored as normal, soft, loose, or watery, and rectal bleeding was scored on a scale of 0–4 based on visual observation or the color development time of the occult blood test. A score of 4 was assigned when the color developed within 10 s, and lower scores were assigned as the development time increased, with 0 points assigned when development occurred after >120 s. After DSS administration, colitis severity was assessed on days 5, 10, 15, 20, 25, and 30, and body weight measurements and fecal sample collection were performed at the corresponding time points.

### 2.5. Morphological Observation of Colon Tissue

Colon tissues from four randomly selected mice were fixed in 4% paraformaldehyde, routinely dehydrated and cleared, and embedded in paraffin. Continuous 4 μm thick sections were cut using a paraffin microtome (SLEE, CUT5062, Mainz, Rheinland Pfalz, Germany) and baked at 60 °C for 30 min. The sections were deparaffinized three times in xylene (Yonghua Chemical, Changshu City, China) for 5–10 min each and then rehydrated through a graded ethanol series (absolute, 95%, and 80% ethanol). The sections were subsequently stained with hematoxylin solution (LEAGENE, DH0006) for 1–5 min. After rinsing with running water, the sections were differentiated with acid alcohol and stained with warm water. The sections were then counterstained with eosin solution (LEAGENE, DH0006) for 5 min. After dehydration and clearing, the sections were mounted on a neutral resin. Histological changes were observed and imaged using a microscope (Olympus, CX33, Tokyo, Japan). Histopathological scoring was performed according to the following criteria: 0, no inflammation; 1, mild inflammation, 0–5% epithelial loss, 0–10% crypt destruction, slight goblet cell reduction, and mild inflammatory cell infiltration; 2, moderate inflammation, 5–10% epithelial loss, 10–20% crypt destruction, moderate goblet cell reduction, and inflammatory cell infiltration; 3, severe inflammation, >10% epithelial loss, >20% crypt destruction, marked goblet cell reduction, and pronounced inflammatory cell infiltration.

### 2.6. Serum Biomarker Analysis

Serum samples from four randomly selected mice were analyzed using ELISA to quantify inflammatory cytokines and oxidative stress marker [25]. The measured indices included IL-6, TNF-α, IL-1β, IL-10, SOD, GSH-Px, and MDA. All assays were performed following the manufacturer’s instructions.

### 2.7. Immunofluorescence Staining

Colon tissues from four randomly selected mice were fixed in 4% paraformaldehyde, embedded in paraffin, and sectioned at a thickness of 4 μm. The sections were deparaffinized, rehydrated, and subjected to antigen retrieval using Tris-EDTA (pH 9.0; Sevilla, G1203, Wuhan, China). Endogenous peroxidase activity was blocked with 3% H_2_O_2_ (Lircon, Dezhou, China) at room temperature for 10 min. After washing with PBS (Solarbio, P1022, Beijing, China), the sections were incubated with blocking solution (Coolaber, SL1330, Beijing, China) at room temperature for 30 min. The sections were then incubated overnight at 4 °C with primary antibodies against ZO-1 (:2000, 21773-1-AP, Proteintech, Wuhan, China), occludin (1:400, Proteintech, 27260-1-AP), Claudin-1 (1:200, Proteintech, 28674-1-AP), and MUC2 (1:800, Proteintech, 27675-1-AP). The following day, after washing with PBS, Alexa Fluor 488/594-conjugated secondary antibody (YEASEN, 33106ES60, Shanghai, China) was added dropwise, and the sections were incubated at 37 °C in the dark for 30 min. The nuclei were counterstained with DAPI (Solarbio, C0060) for 5 min. After washing with PBS, the sections were mounted using an anti-fade medium. Fluorescence images were acquired using an Olympus CX33 fluorescence microscope, and the mean fluorescence intensity was quantified using the Image-Pro Plus 6.0 software.

### 2.8. Fecal Metagenomic Analysis

In fecal metagenomic testing, DNA was not successfully extracted in two mice in the PC-01 group, and there were no excess samples. The final sample size was 36, with six mice in each group. Raw sequencing data were first subjected to quality control using fastp (v0.20.0) [26], and mouse host-derived sequences were removed using BWA (v0.7.17) [27]. Clean reads were then assembled using MEGAHIT (v1.1.2) [28], and open reading frames (ORFs) were predicted using Prodigal (v2.6.3) [29]. Sequences were clustered using CD-HIT (v4.6.1) at an identity threshold of ≥90.0% and a coverage threshold of ≥90.0% to generate a non-redundant gene catalog. High-quality reads were subsequently aligned to the gene catalog using SOAP 2.21aligner at an identity threshold of ≥95.0% to estimate species abundance. Species-level and functional annotations were assigned using DIAMOND (v2.0.13) against the NR and KEGG databases, and carbohydrate-active enzymes were annotated using HMMER (v3.1b2) [30,31].

Statistical analyses were performed using Python (v2.7.0) and R (4.4.3). Alpha diversity indices and beta diversity analyses were both calculated using R (4.4.3). Microbial beta diversity was assessed using the Bray–Curtis distance, and differences in community structure were evaluated using principal coordinate analysis (PCoA) and analysis of similarity (ANOSIM).

### 2.9. qPCR Analysis

Total RNA was extracted from the colon tissues of four randomly selected mice using TRIzol reagent (Xinbaiji, Chengdu, China) in combination with an automated magnetic-bead extraction protocol performed on an M1 extractor. RNA purity was assessed using a Unano-1000 analyzer, and samples that met the quality requirements were reverse-transcribed into cDNA using the FUTURE Script 1st Strand cDNA Synthesis Kit (Aidlab, Beijing, China). Quantitative PCR was performed on a Borui FQD-96C real-time fluorescence quantitative analyzer using the SYBR Green Supermix system (Beijing Edlai, Beijing, China). The amplification program consisted of initial denaturation at 95 °C for 3 min, followed by 40 cycles at 95 °C for 10 s and 60 °C for 30 s, with subsequent melting curve analysis. Actb was used as the internal reference gene, and relative target gene expression was calculated using the 2^−ΔΔCt^ method [32]. The primer sequences are provided in Appendix A.

### 2.10. Statistical Analysis

Graphing and statistical analyses were conducted using R (4.4.3) and OriginPro (2026), and the figures were organized and formatted using Adobe Illustrator (2026). All data are presented as mean ± standard deviation (SD). Intergroup differences were analyzed using the non-parametric Wilcoxon rank-sum test, and *p* values for multiple comparisons were adjusted using the Benjamini–Hochberg procedure to control the false discovery rate (FDR). The R packages vegan (v.2.5-1) and ggpubr were used to calculate alpha diversity of the gut microbiota. Principal coordinate analysis (PCoA) based on the Bray–Curtis distance was performed to evaluate changes in the microbial community structure. ANOSIM was used to assess the overall differences in gut microbiota structure among the groups. A *p* value < 0.05 was considered statistically significant.

## 3. Results

### 3.1. Heat-Inactivated PC-01 Significantly Alleviates Disease Severity in DSS-Treated Mice

Throughout the experiment, the DAI score in the NC group remained low and stable, ranging from 0 to 2. After DSS administration, mice in the DSS group developed loose stools and fecal occult blood, accompanied by a marked increase in the DAI score (Figure 1B) and colon length was shortened (Figure 1C). Colon weight-to-length ratio were significantly lower in the DSS group than in the NC group (Figure 1C, *p* < 0.01). In contrast, treatment with heat-inactivated PC-01 significantly decreased the DAI score in DSS-treated mice (Figure 1B) and partially restored the colon length compared to the DSS group (Figure 1C). These findings indicate that heat-inactivated PC-01 effectively attenuates DSS-induced colitis and promotes recovery.

### 3.2. Effects of Heat-Inactivated PC-01 on Colon Histopathology and Intestinal Mucosal Barrier Function in Mice

H&E staining of colon tissue is a widely used method for evaluating tissue injury. The histopathological changes in each group are shown in Figure 1D. Compared with the NC group, mice in the DSS group exhibited severe colon damage, including marked disruption of tissue architecture, mucosal ulceration, loss of intestinal glands, inflammatory cell infiltration in the mucosa, and a reduced number of goblet cells. In contrast, the overall colonic structure in the PC-01 group was largely preserved, with orderly intestinal glands and no obvious reduction in the number of goblet cells. Intestinal mucosal injury was substantially alleviated, with only minor surface irregularities (Figure 1D). Quantitative analysis of tissue injury further showed that both the DSS and PC-01 groups exhibited intestinal damage relative to the NC group; however, the severity of injury in the PC-01 group was significantly lower than that in the DSS group (Figure 1D, *p* < 0.05). These results suggest that PC-01 heat-inactivated bacterial effectively mitigates DSS-induced damage to the colon tissue.

In addition, DSS caused pronounced injury to the intestinal mucosa and reduced the distribution and expression of mucosal barrier-related proteins, including MUC2, Occludin, Claudin-1, and ZO-1. Following treatment with heat-inactivated PC-01, the distribution and expression of MUC2, Occludin, Claudin-1, and ZO-1 showed numerical increases to varying degrees compared with the DSS group. However, these changes were not statistically significant. In particular, the expression levels of Occludin and Claudin-1 in the PC-01 group were approximately 1.5-fold and 1.3-fold higher, respectively, than those in the DSS group. Therefore, these results indicate a numerical trend toward improved colonic barrier-related protein expression.

### 3.3. Heat-Inactivated PC-01 Regulates Serum Cytokine Levels and Oxidative Stress

To evaluate the regulatory effect of PC-01 on the inflammatory status of mice, we measured the serum levels of key cytokines. Compared with the NC group, DSS treatment significantly increased the serum levels of the pro-inflammatory cytokines IL-6, IL-1β, and TNF-α (Figure 2A, *p* < 0.001) and significantly decreased the level of the anti-inflammatory cytokine IL-10 (Figure 2A, *p* < 0.0001). Intervention with PC-01 heat-inactivated bacterial significantly suppressed IL-6 (*p* < 0.01), IL-1β (*p* < 0.001), and TNF-α (*p* < 0.01) levels, and markedly increased IL-10 levels (*p* < 0.001). These results indicate that the administration of heat-inactivated PC-01 effectively reduced systemic pro-inflammatory cytokine levels and enhanced the anti-inflammatory response in mice.

The inflammatory response induced by DSS can also trigger oxidative stress through excessive production of reactive oxygen species, thereby damaging the intestinal mucosal barrier. Compared to the NC group, the DSS group showed significantly lower levels of GSH-Px and SOD and a significantly higher level of MDA (Figure 2B, *p* < 0.01). Treatment with heat-inactivated PC-01 improved oxidative stress status, significantly increasing GSH-Px and SOD levels (*p* < 0.01) and significantly decreasing MDA levels (*p* < 0.01). These findings suggest that heat-inactivated PC-01 effectively ameliorates DSS-induced oxidative stress.

### 3.4. Heat-Inactivated PC-01 Suppresses Activation of Intestinal Inflammatory Pathways

Based on qPCR analysis of genes related to the NF-κB and NLRP3 inflammasome pathways in mouse intestinal epithelial cells, the results showed that the expression levels of TLR4, NLRP3, CASP1, IL-1β, Nfkb1, Rela, MCP-1, and Cxcl10 in the colon tissues of DSS-induced mice exhibited an increasing trend. Intervention with heat-inactivated PC-01 reduced the expression of TLR4, NLRP3, CASP1, IL-1β, Nfkb1, Rela, MCP-1, and Cxcl10. Although these changes did not reach statistical significance (Figure 3, *p* > 0.05), the overall expression pattern demonstrated clear biological relevance.

### 3.5. Heat-Inactivated PC-01 Modulates Gut Microbiota Structure and Restores Species Composition

To evaluate the effect of heat-inactivated PC-01 on the gut microbiota, we performed alpha and beta diversity analyses of fecal samples from each group. Alpha diversity analysis showed that DSS treatment significantly increased the Shannon index (*p* < 0.01) and significantly decreased the Simpson index (*p* < 0.05). In contrast, no significant changes in alpha diversity indices were observed in the PC-01 group (Figure 4A, *p* > 0.05). PCoA based on Bray–Curtis distances revealed a clear separation among the NC, DSS, and PC-01 groups (R = 0.516, *p* = 0.001). Samples from the PC-01 group were positioned between those from the NC and DSS groups, indicating that DSS markedly altered the gut microbial structure, whereas heat-inactivated PC-01 altered the microbial community composition.

We further analyzed the alterations in gut microbiota composition at the species level. Compared with the NC group, DSS disrupted the gut microbial composition of mice, as reflected by a significant reduction in the relative abundances of *Barnesiella* and *Muribaculum* and a significant enrichment of *Angelakisella*, *Lawsonibacter*, and other taxa (*p* < 0.01). Heat-inactivated PC-01 significantly increased the relative abundances of *Barnesiella* and *Muribaculum* and reduced the relative abundances of *Angelakisella*, *Lawsonibacter*, and other taxa (*p* < 0.05). These findings suggest that these bacteria may be key microbial targets through which PC-01 alleviates DSS-induced intestinal inflammation and oxidative stress.

### 3.6. Heat-Inactivated PC-01 Modulates DSS-Associated Gut Microbial Functional Profiles

Changes in the abundance and composition of the gut microbiota can affect its function. Compared with the NC group, the gut microbiota of DSS-treated mice showed significant upregulation of ribosome biogenesis and fatty acid elongation pathways (Figure 5A, *p* < 0.05). In contrast, the TCA cycle, riboflavin metabolism, Toll and Imd signaling pathways, and lipopolysaccharide biosynthesis pathway were significantly suppressed (Figure 5A, *p* < 0.05). Intervention with heat-inactivated PC-01 reduced the relative abundance of microbial genes assigned to ribosome biogenesis and fatty acid elongation, partially shifting the corresponding microbial functional profiles toward those of the NC group. Moreover, the TCA cycle, riboflavin metabolism, Toll and Imd signaling pathways, and lipopolysaccharide biosynthesis pathway, which were markedly inhibited in the DSS group, were significantly enriched after PC-01 treatment compared to the DSS group (Figure 5A, *p* < 0.05). These pathways are closely associated with energy metabolism, lipid metabolism, inflammatory responses, and pathogen-related molecular recognition.

Functional annotation of intestinal carbohydrate-active enzymes based on the CAZy database showed similar results. The CAZymes identified in the mouse gut mainly included auxiliary activities (AA), glycoside hydrolases (GH), carbohydrate-binding modules (CBM), carbohydrate esterases (CE), glycosyltransferases (GT), polysaccharide lyases (PL), and S-layer homology modules (SLH), among other CAZy-related functional categories (Figure 5B). Compared with the NC group, the DSS group showed an overall increase in the relative abundances of AA, PL, and SLH, whereas the relative abundances of GH, GT, and CE decreased. Heat-inactivated PC-01 partially reversed these trends, as evidenced by the reduced relative abundances of AA, PL, and SLH and increased relative abundances of GH, GT, and CE compared with the DSS group (Figure 5D). Heatmap analysis (Figure 5C) revealed distinct abundance distribution patterns among the three groups at the level of the major CAZy families. Heat-inactivated PC-01 significantly increased the relative abundance of several CAZy families, including GH13 and GT19, and other families involved in carbohydrate degradation and transformation, while reducing the relative abundance of certain abnormally enriched families. Collectively, these findings indicate that heat-inactivated PC-01 partially modulates DSS-associated changes in the functional potential of the gut microbiota, as reflected by alterations in KEGG-annotated pathways and CAZy-associated gene profiles.

## 4. Discussion

IBD is a chronic, recurrent, and immune-mediated intestinal disorder. Accumulating evidence indicates that gut dysbiosis profoundly influences the pathogenesis of IBD. Although conventional probiotics are widely used, their heat-inactivated forms have demonstrated notable anti-inflammatory effects and may offer advantages in terms of safety and stability. Therefore, this study evaluated the ameliorative effect of heat-inactivated PC-01 on DSS-induced colitis in mice and its impact on intestinal immune responses. The experimental results showed that heat-inactivated PC-01 enhanced the integrity of the intestinal epithelial barrier, augmented systemic anti-inflammatory and antioxidant capacities, and ameliorated DSS-induced symptoms. These protective effects appear to arise from the modulation of the gut microbiota and metabolic profile, thereby alleviating colitis.

The DAI, colon length, and histopathological damage to the colon are key parameters for evaluating DSS-induced colitis severity. Previous studies have demonstrated that PC-01 effectively ameliorates DSS-induced intestinal inflammation. The current findings further confirm that treatment with heat-inactivated PC-01 significantly reduced the DAI score in DSS-treated mice and attenuated the colon tissue damage. Heat-inactivated bacteria, like their viable counterparts, exhibit beneficial effects, possibly because their cellular and molecular components directly interact with the gastrointestinal mucosa. Such interactions stimulate pattern recognition receptors (e.g., Toll-like receptors, TLRs) and exert immunoregulatory and anti-inflammatory effects in the intestine. Importantly, these effects are independent of bacterial colonization and are mediated by bacterial structural components (such as cell wall molecules) [33]. Previous reports have indicated that inactivated probiotics can modulate intestinal inflammation and immune function. For example, Lee et al. showed that heat-inactivated *Lacticaseibacillus paracasei* HY2782 reduced DAI scores, mitigated weight loss and colon shortening, improved histopathological features, and suppressed pro-inflammatory cytokine expression [34]. Zhong et al. demonstrated that heat-inactivated *Companilactobacillus crustorum* MN047 (CC) alleviates pathological changes in ulcerative colitis, lowers the disease activity index, and prevents colon shortening [35]. These results suggest that probiotic inactivation is an effective strategy for alleviating IBD and that PC-01 is a promising candidate strain for ameliorating related symptoms.

Excessive release of pro-inflammatory cytokines and elevated oxidative stress are hallmarks of DSS-induced colitis [36]. In this pathological process, disruption of the intestinal epithelial barrier is a critical initiating event that triggers inflammation [37]. DSS disrupts epithelial integrity, allowing luminal antigens to invade and initiate an immune inflammatory cascade. Throughout this process, changes in intestinal permeability and loss of mucosal barrier proteins (e.g., such as tight junction proteins) are key indicators of inflammation severity and therapeutic efficacy [38]. Heat-inactivated PC-01 was associated with improvements in mucosal morphology and numerical increases in the expression and distribution of the tight-junction proteins Occludin and Claudin-1. However, these changes were not statistically significant. Thus, the present findings suggest a potential beneficial effect of heat-inactivated PC-01 on intestinal epithelial barrier-related responses rather than providing conclusive evidence of barrier restoration. These trends were consistent with the observed improvements in inflammatory and oxidative stress markers. For example, heat-inactivated PC-01 reduced the levels of IL-6, IL-1β, TNF-α, and MDA, while increasing the levels of IL-10, SOD, and GSH-Px. TNF-α, IL-6, and IL-1β are typical pro-inflammatory cytokines that promote inflammatory cell recruitment, exacerbate epithelial damage, and sustain local inflammation [39]. IL-1β, the end product of NLRP3 inflammasome activation, is typically released during Caspase-1-mediated pyroptosis, exacerbating colonic necrosis and ulceration [40]. IL-10 is a key anti-inflammatory cytokine in the intestine that is critically involved in modulating inflammatory signaling [41]. SOD and GSH-Px are essential antioxidant enzymes that scavenge reactive oxygen species (ROS) and maintain redox homeostasis. By clearing excess ROS and reducing MDA levels, these enzymes mitigate oxidative stress-induced damage to intestinal epithelial cells and mucosal barriers [42]. Collectively, these findings suggest that heat-inactivated PC-01 mitigates DSS-induced inflammation and oxidative stress by inhibiting NF-κB pathway activation and NLRP3 inflammasome activation. This conclusion is further supported by gene expression analysis, which showed the downregulation of colon tissue genes such as Nfkb1, Rela, chemokines MCP-1 and Cxcl-10, Casp1, and IL-1β.

The gut microbiota is a key regulator of host immune homeostasis and intestinal barrier integrity and plays a decisive role in the pathogenesis and resolution of intestinal inflammation [43]. In DSS-treated mice, heat-inactivated PC-01 was associated with an attenuation of gut microbiota dysbiosis, characterized by increased relative abundances of genera generally considered beneficial, including *Barnesiella* and *Muribaculum*, and decreased relative abundances of *Angelakisella* and *Lawsonibacter*. These compositional changes coincided with the improvement in colitis-related outcomes observed in the PC-01-treated group, suggesting that modulation of the gut microbiota may be involved in the protective effects of PC-01. Previous studies have provided a potential biological context for these observations. For example, Liu et al. reported that *Barnesiella* was associated with increased colonic expression of the tight junction proteins ZO-1 and Occludin and reduced TNF-α expression in an experimental setting [44]. In addition, *Muribaculum*, a commensal gut genus, has been reported to be associated with the maintenance of epithelial barrier integrity. Notably, *Muribaculum intestinale* alleviates intestinal inflammation by suppressing excessive TLR4/MyD88/NF-κB signaling, accompanied by reduced expression of TNF-α, IL-6, and IL-1β and restoration of tight junction proteins such as ZO-1 [45,46]. In contrast, *Angelakisella* and *Lawsonibacter* have been associated with inflammatory conditions. *Angelakisella* may interact with host- or microbiota-derived neurotransmitter-related compounds and influence the neuroimmune axis [47], whereas an increased abundance of *Lawsonibacter* has been associated with the activation of inflammatory pathways, including the NLRP3 inflammasome pathway [48]. Thus, the amelioration of DSS-induced colitis by PC-01 appears to be linked to its modulation of these key gut microbial taxa.

Gut microbiota dysbiosis inherently alters the functional pathways of microbes. In this study, heat-inactivated PC-01 significantly reversed DSS-induced disruptions in microbial metabolism, notably restoring pathways related to energy and riboflavin metabolism. Energy deficiency in intestinal epithelial cells is thought to contribute to IBD pathogenesis, as a sufficient ATP supply is crucial for maintaining the barrier. For instance, the TCA cycle intermediate α-ketoglutarate (α-KG) can inhibit the activation of IKKβ, a key mediator of the pro-inflammatory NF-κB pathway [49]. α-KG also serves as a precursor for glutamine and glutamate, making it an important energy source for intestinal cells and essential for protecting the mucosal barrier [50]. In addition, Zhong et al. [51] found that DSS-induced inflammation dysregulates riboflavin metabolism, potentially enhancing host immune activation and aggravating mucosal inflammation. CAZymes are important components of gut microbial function [52]. DSS-induced colitis disrupted the CAZyme profile: the relative abundance of enzymes involved in complex carbohydrate degradation (such as GHs and CEs) decreased, whereas others (such as AAs, PLs, and SLHs) increased. This shift suggests that DSS treatment alters the utilization of carbohydrate substrates by the gut microbiota. Treatment with PC-01 reshaped the intestinal CAZyme profile in mice, restoring the levels of GTs, GHs, and CEs and reducing the levels of AAs and SLHs. GHs hydrolyze glycosidic bonds in dietary polysaccharides and host glycans, facilitating their degradation, and CEs remove side-chain modifications from polysaccharides to further promote their breakdown [53]. GTs catalyze glycosyl transfer reactions and are central to glycosylation processes. Alterations in glycosylation are closely associated with intestinal diseases. IBD research has highlighted changes in glycosylation patterns and their relationship with mucosal function [54]. Abnormal glycosyltransferase activity has been observed in colorectal cancer, suggesting that GT-related glycosylation processes may contribute to intestinal pathologies [55]. Therefore, PC-01’s regulation of CAZyme-related functions may help restore the metabolic network of the gut microbiota, thereby supporting intestinal barrier repair and alleviating inflammation.

Nevertheless, this study has certain limitations in terms of mechanistic interpretation. Although the mRNA expression of key genes associated with the TLR4/NF-κB/NLRP3 signaling pathway, these results provide transcriptional associations rather than direct evidence of pathway activation. Protein-level validation, such as the expression of TLR4, NF-κB p65, NLRP3, and cleaved caspase-1. In the following experiments, we will combine pathway-related proteins to explore the underlying mechanism.

## 5. Conclusions

In conclusion, this study demonstrated that heat-inactivated PC-01 significantly ameliorated DSS-induced colitis in mice. Its protective effects are associated with modulation of the intestinal microbiota and related metabolic pathways, enhancement of intestinal barrier function, and suppression of inflammation and oxidative stress, ultimately reducing disease severity. These findings provide new insights into potential intervention strategies for IBD, establish an experimental foundation and theoretical basis for the future clinical translation of PC-01, and support the development of heat-inactivated PC-01 for incorporation into ambient-stable dairy products, thereby expanding its potential application.

## Figures and Tables

**Figure 1:**
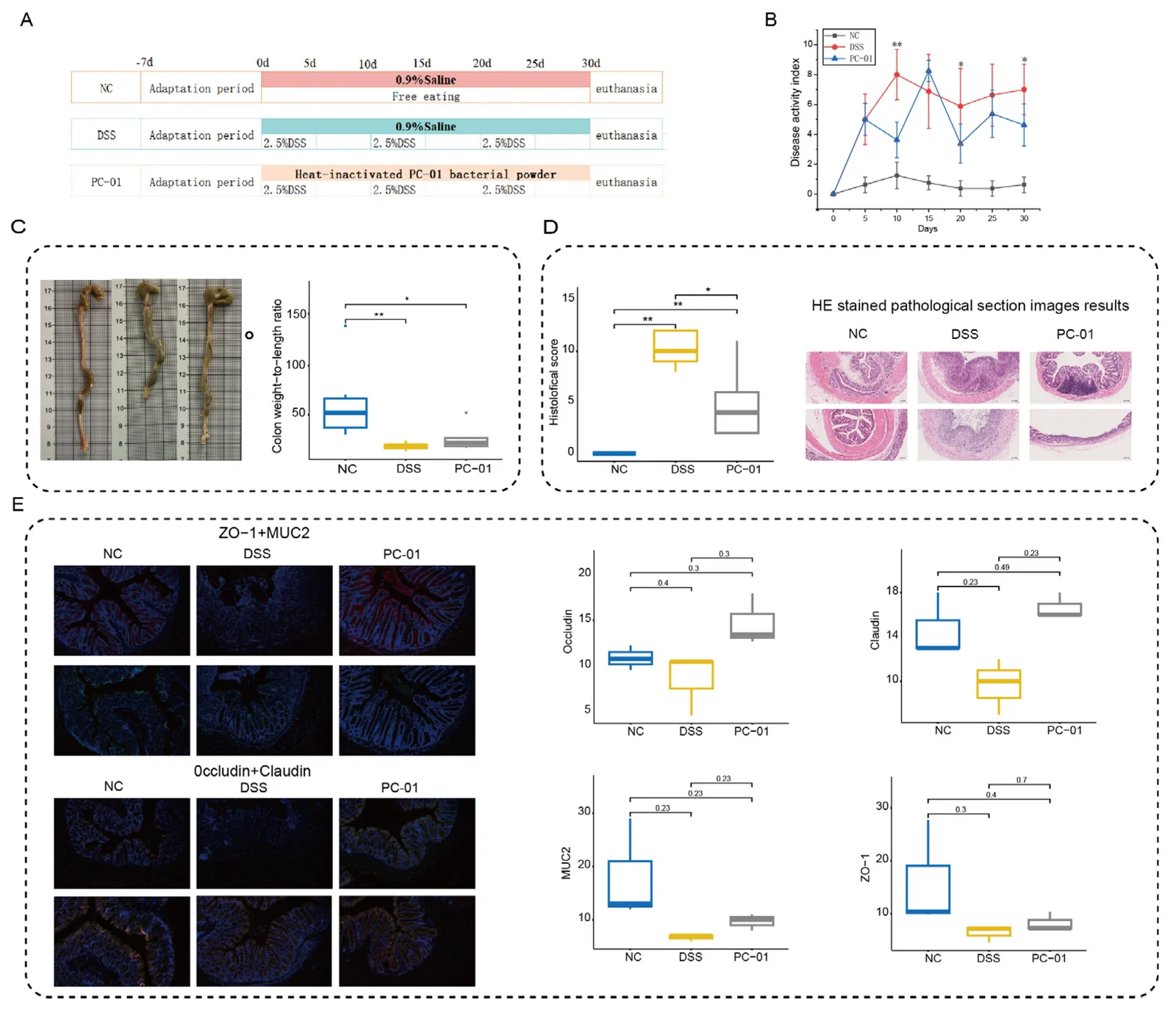
Heat-inactivated PC-01 alleviates DSS-induced colitis and improves intestinal barrier injury in mice. (**A**) Flowchart of animal experiment design; (**B**) DAI score (* for comparison between DSS group and PC-01 group, error bars: SD, n = 8). (**C**) Colon length, colon length to weight ratio; (**D**) HE staining score and mouse HE staining pathological section results (n = 8); (**E**) ZO-1, MUC2, Occludin, Claudin-1 distribution map, Occludin expression level, Claudin-1 expression level, MUC2 expression level, ZO-1 expression level (n = 4). Group information: NC: Control group; DSS: Model Group; PC-01: Heat-inactivated PC-01 group. Statistical significance was assessed using the Wilcoxon rank-sum test, and *p* values were adjusted for multiple comparisons using the false discovery rate (FDR) method, **: *p* < 0.01, *: *p* < 0.05.

**Figure 2:**
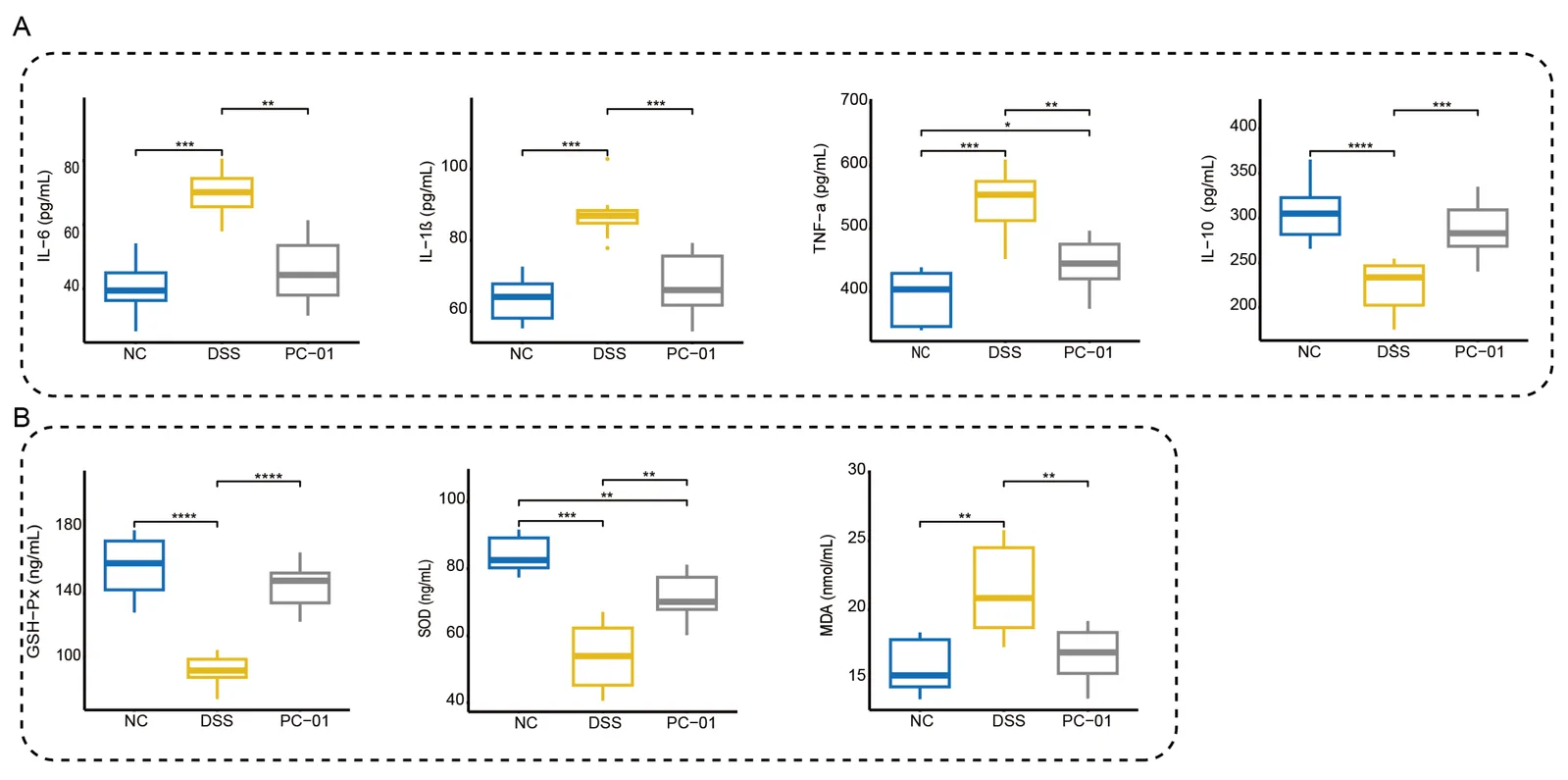
Effect of heat-inactivated PC-01 on inflammatory factors and oxidative stress levels in DSS-induced colitis mice. (**A**) Inflammatory cytokine levels in mouse serum (IL-6, IL-1β, TNF-α, IL-10). (**B**) Oxidative stress levels in mouse serum (GSH-PX, SOD, MDA,). Group information: NC: Control group; DSS: Model Group; PC-01: Heat-inactivated PC-01 group; n = 4. Statistical significance was assessed using the Wilcoxon rank-sum test, and *p* values were adjusted for multiple comparisons using the false discovery rate (FDR) method. ****: *p* < 0.0001, ***: *p* < 0.001, **: *p* < 0.01, *: *p* < 0.05.

**Figure 3:**
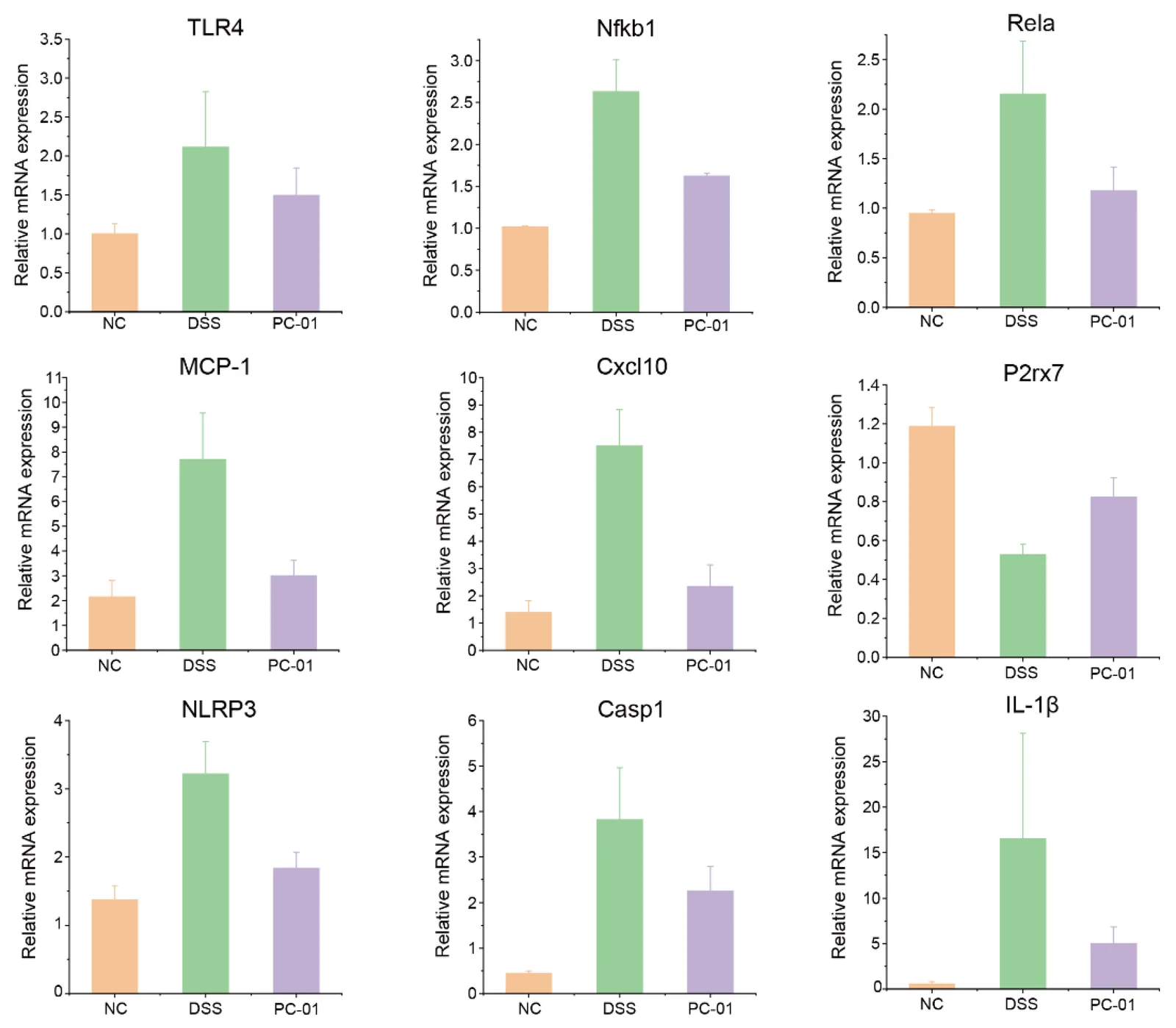
Effects of qPCR on the Intestinal Transcriptome of Mice in Each Group. Grouping information: NC: Control group; DSS: Model Group; PC-01: Heat-inactivated PC-01 Group; error bars: SD, n = 4. Statistical significance was assessed using the Wilcoxon rank-sum test, and *p* values were adjusted for multiple comparisons using the false discovery rate (FDR) method.

**Figure 4:**
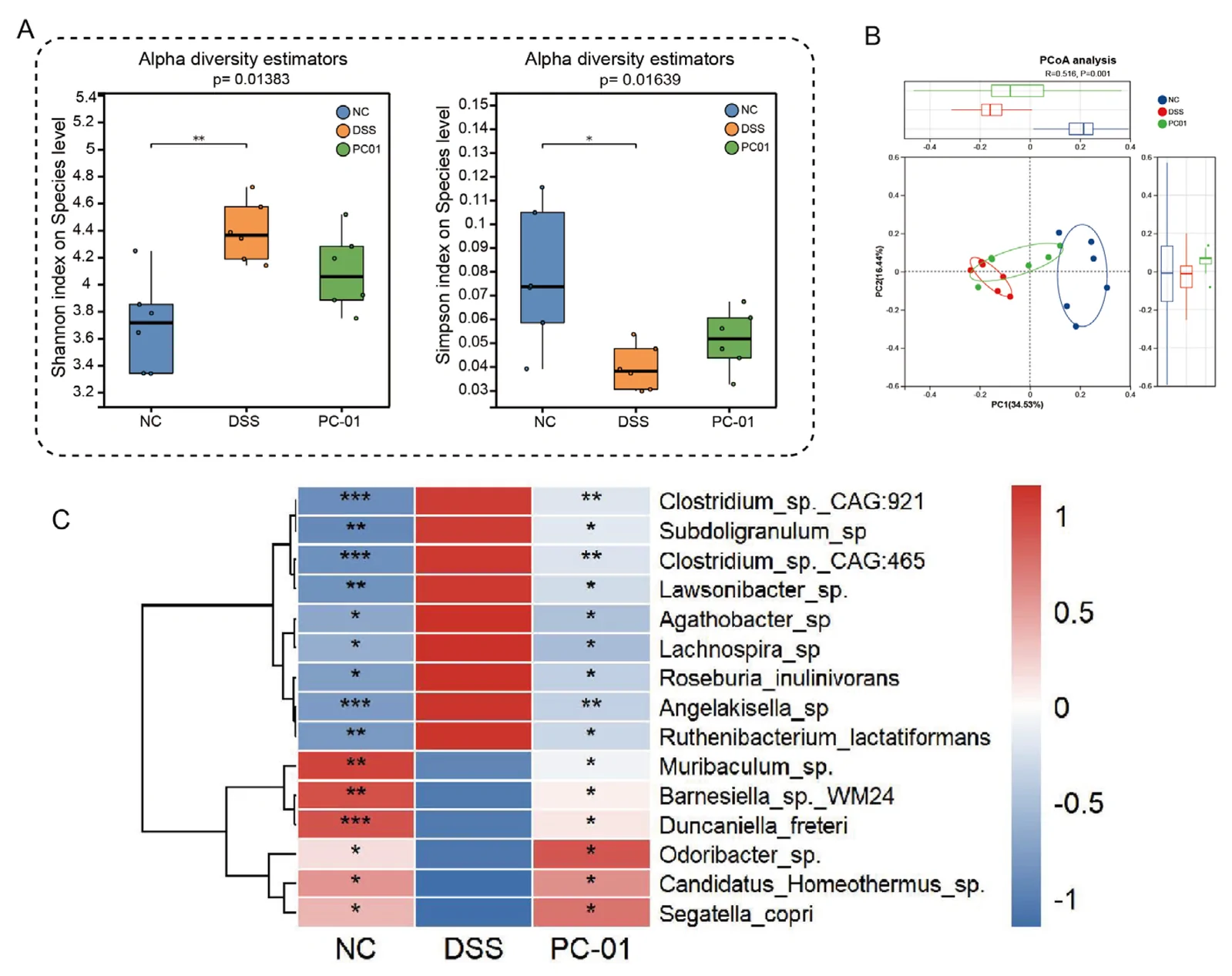
Effect of heat-inactivated PC-01 on gut microbiota in DSS-induced colitis mice. (**A**) Analysis of alpha diversity in gut microbiota of mice in each group, including Shannon index and Simpson index. (**B**) Based on PCoA analysis of β diversity, the R and *p* values in the figure are derived from ANOSIM analysis. (**C**) Thermogram of relative abundance of gut microbiota species. Group information: NC: Control group; DSS: Model Group; PC-01: Heat-inactivated PC-01 Group; n = 6, ***: *p* < 0.001,**: *p* < 0.01, *: *p* < 0.05.

**Figure 5:**
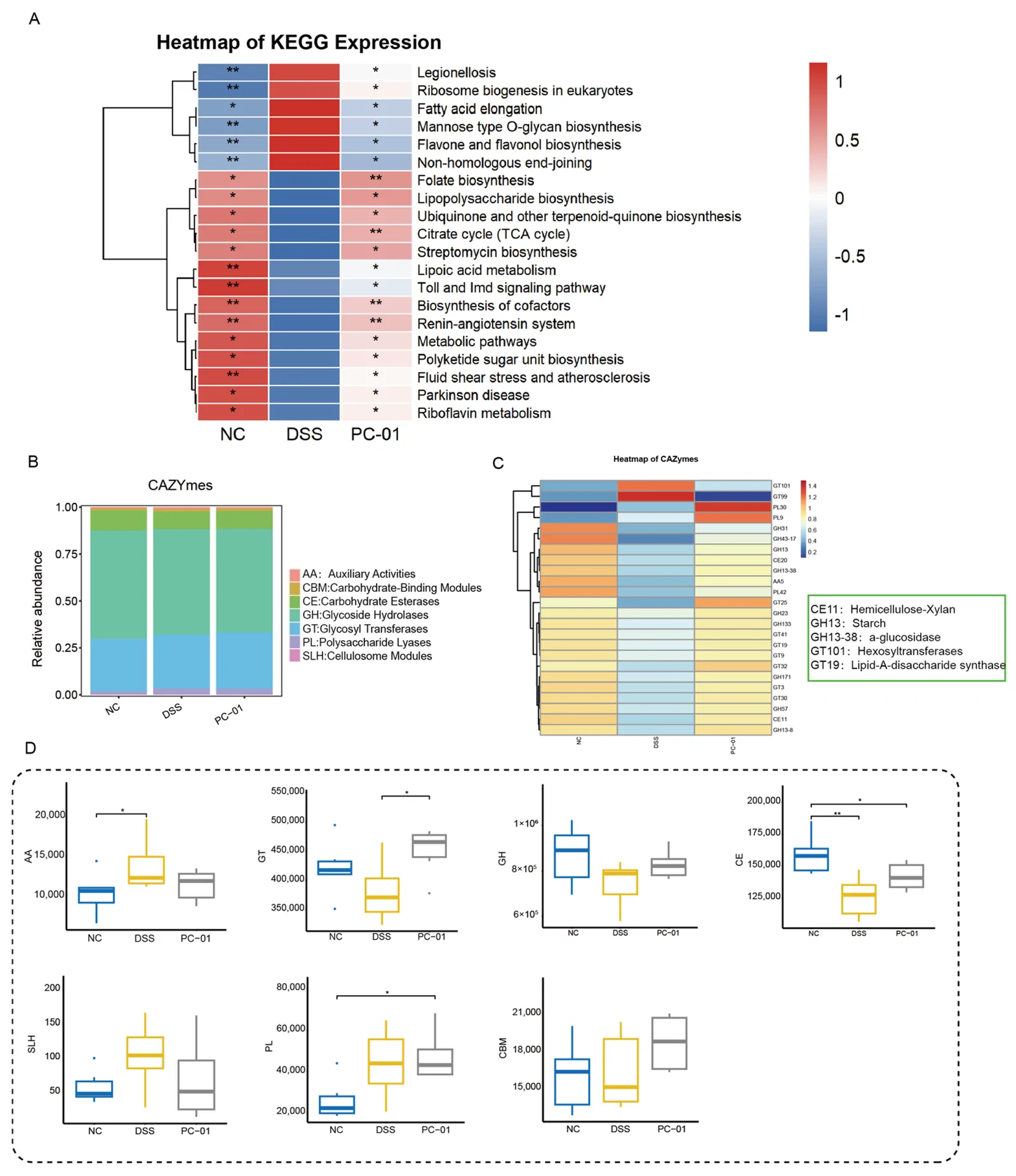
Effect of heat-inactivated PC-01 on gut microbiota function in DSS-induced colitis mice. (**A**) KEGG functional pathway enrichment. (**B**) Classification and statistics of carbohydrate active enzymes. (**C**) Thermographic analysis of carbohydrate enzymes in the gut microbiota of mice in each group. (**D**) Significance analysis of relative abundance of various genes related to enzymes in each group of mice; Group information: NC: Control group; DSS: Model Group; PC-01: Heat-inactivated PC-01 group; n = 6. Statistical significance was assessed using the Wilcoxon rank-sum test, and *p* values were adjusted for multiple comparisons using the false discovery rate (FDR) method. **: *p* < 0.01, *: *p* < 0.05.

## Data Availability

The datasets generated and/or analyzed in the current study are available in the National Genomics Data Center (NGDC) repository. The data can be accessed using the accession number CRA046377 (https://ngdc.cncb.ac.cn/gsa/s/Bh4gy33W, accessed on 19 September 2026).

## Appendix A

**Table A1:** Primer information.

Allele Number	Primer Name	Sequence (5′ to 3′)
internal reference	β-actin-F	GTCCCTCACCCTCCCAAAAG
	β-actin-R	GCTGCCTCAACACCTCAACCC
1	1-Nlrp3-F	CCAAGGAGGAAGAAGAAGAGA
	1-Nlrp3-R	TAGAAGTGAGGCAGCAGTT
2	2-Casp1-F	GAATATGCCTGGTCTTGTGA
	2-Casp1-R	CGGGAAGAGGTAGAAACG
3	3-Il1b-F	CTTCAGGCAGGCAGTATC
	3-Il1b-R	CCAGCAGGTTATCATCATCA
4	4-P2rx7-F	GCTGTGTCCTGAGTATCC
	4-P2rx7-R	GGTCTTCCTTGTCTTGTCA
5	5-Tlr4-F	TCACCTCTGCCTTCACTAC
	5-Tlr4-R	GACACTACCACAATAACCTTCC
6	6-Nfkb1-F	CAGAAGACACGAGGCTACA
	6-Nfkb1-R	CGGAAGGCATTGTTCAGTATC
7	7-Rela-F	CAGACCCAGGAGTGTTCA
	7-Rela-R	TGTCACCAGGCGAGTTAT
8	8-MCP-1-F	CACCTGCTGCTACTCATTC
	8-MCP-1-R	GCTTCTTTGGGACACCTG
9	9-Cxcl10-F	AGTGGGACTCAAGGGATC
	9-Cxcl10-R	GCTCGCAGGGATGATTTC
